# Chronic Headache and Visual Field Loss: Manifestations in a Patient With Chronic *Brucellosis* Meningitis: Case Report With Brief Literature Review

**DOI:** 10.1155/crdi/8192652

**Published:** 2026-07-06

**Authors:** Shiva Shabani

**Affiliations:** ^1^ Department of Infectious Diseases, School of Medicine, Arak University of Medical Sciences, Arak, Iran, arakmu.ac.ir

**Keywords:** chronic *brucellosis* meningitis, chronic headache, visual field loss

## Abstract

Brucellosis is a zoonotic infection caused by *Brucella* species, often leading to serious complications and presenting diagnostic challenges. A 45‐year‐old female patient presented with a headache that had persisted for two months and was recently accompanied by visual disturbances. Notably, the patient’s headache began 6 weeks postpartum, and she had no prior history of illness, medication use, or headaches. Imaging studies were normal, and cerebrospinal fluid (CSF) analysis revealed meningitis, with a positive Wright test. After excluding other potential causes and considering the patient’s consumption of local dairy products, the diagnosis of *Brucella* meningitis was confirmed. The patient was treated with a standard three‐drug regimen for 6 months and was followed throughout the treatment period. Initially, her headache resolved, followed by improvement in visual symptoms. Follow‐up serologic tests showed a declining trend in *Brucella* titers, and perimetry results returned to normal by the fourth month of treatment. This case underscores the importance of including *Brucella* in the differential diagnosis of patients with chronic meningitis, especially in endemic regions, and highlights the favorable outcomes associated with prompt treatment.

## 1. Introduction

*Brucella* is a genus of Gram‐negative, intracellular, aerobic bacteria comprising six distinct species, four of which are pathogenic to humans. This complex infectious disease has a multifaceted impact on the body, affecting various systems including the liver, spleen, bone marrow, lymph nodes, nervous system, musculoskeletal system, cardiovascular system, gastrointestinal tract, and genitourinary system. Brucellosis is among the most prevalent zoonotic diseases worldwide, with more than 500,000 new cases reported annually. The disease is particularly endemic in the Mediterranean region and parts of the Middle East, including countries such as Syria, Iraq, and Iran. Human transmission primarily occurs through direct contact with infected animals—such as cattle and sheep—or indirectly through the consumption of undercooked meat and unpasteurized dairy products [1].

Patients with brucellosis may present with a broad spectrum of signs and symptoms, including fever, fatigue, weakness, excessive sweating, joint pain, decreased appetite, headache, myalgia, lower back pain, hepatosplenomegaly, and arthritis [2]. When the nervous system is involved, additional neurological manifestations may occur, such as meningitis, meningoencephalitis, encephalitis, cranial nerve dysfunction, increased intracranial pressure, cerebral vasculitis, radiculitis, peripheral neuropathy, and myelitis. Psychiatric symptoms—including depression, anxiety, and psychosis—may also be observed [3].

Neurobrucellosis (NB) is a rare complication of brucellosis, with an incidence of less than 10% among affected patients. The onset is often insidious, typically occurring 2–12 months after initial exposure, and may present atypically. This can lead to diagnostic delays and confusion with other infections, such as tuberculosis [4]. Although the underlying mechanisms of NB remain unclear, three hypotheses have been proposed: (a) a direct neuropathic effect, (b) the release of cytokines or endotoxins, and (c) an inflammatory or immune‐mediated response to *Brucella* within the nervous system. The bacteria may enter the bloodstream through the reticuloendothelial system, resulting in bacteremia and potential invasion of the meninges [5]. Because the clinical manifestations of NB are diverse and lack specific distinguishing features, timely and accurate diagnosis is essential to initiate appropriate treatment and minimize long‐term complications [6].

## 2. Case Presentation

A 45‐year‐old female patient presented with a two‐month history of headaches that began approximately six weeks after giving birth. Initially, she attributed the headaches to normal postpartum recovery and did not seek medical attention. Over time, the headaches became nearly constant, and she reported no prior history of similar pain. Approximately 1 week before her consultation, she also developed visual disturbances in her left eye.

On initial examination, the patient was alert with stable vital signs. A comprehensive cranial nerve assessment yielded normal results, and no abnormalities were detected in the peripheral nerve examination or deep tendon reflexes. The patient denied neck pain or photophobia, and no signs of neck stiffness were observed.

Funduscopic examination, however, revealed papilledema in the left eye, indicating increased intracranial pressure. This finding was accompanied by a reduction in the visual field of the left eye. Ophthalmologic evaluation ruled out ocular causes such as retinal detachment or glaucoma. The findings were instead suggestive of optic nerve involvement, most consistent with elevated intracranial pressure secondary to *Brucella* meningitis.

Given the chronicity of the patient’s headaches and the abnormal neurological findings, further diagnostic evaluation was warranted. Imaging studies and cerebrospinal fluid (CSF) analysis were performed to assess the patient’s symptoms and exclude other serious conditions.

Magnetic resonance imaging (MRI) of the brain, performed with and without contrast, demonstrated normal intracranial anatomy, appropriate gray–white matter differentiation and no evidence of mass lesions or tumors. The cerebral hemispheres, cerebellum, and brainstem exhibited normal signal characteristics. The basal ganglia and ventricular system were unremarkable, midline structures were appropriately positioned, and both the internal carotid and basilar arteries were patent with no abnormal enhancement. Magnetic resonance venography (MRV) confirmed patency of all major venous sinuses, with no evidence of superior sagittal sinus thrombosis.

CSF polymerase chain reaction (PCR) testing for *Cryptococcus*, *Mycobacterium tuberculosis*, *Candida*, and *Aspergillus* was negative. CSF VDRL, as well as smear and culture for *Mycobacterium tuberculosis*, were also negative. Serum Wright, 2‐mercaptoethanol (2 ME), and Coombs Wright tests were positive, with titers of 1:160, 1:80, and 1:160, respectively. In contrast, the CSF Wright test demonstrated a higher titer of 1:320. Additional laboratory tests—including rheumatoid factor (RF), antinuclear antibodies (ANAs), VDRL, and HIV—were negative, and the angiotensin‐converting enzyme (ACE) level was within normal limits. Imaging studies, including chest and abdominal CT scans, mammography, breast ultrasound, and abdominopelvic ultrasound, were unremarkable (Table 1).

**TABLE 1:** Laboratory test results and reference ranges.

Test	Result	Normal range
CSF protein	15	15–45 mg/dL
CSF glucose	60	50–75 mg/dL
CSF WBC count	20	0–5 cell/μL
CSF Wright	1/320	< 1:80
(PCR) testing of the CSF for *Cryptococcus*, *tuberculosis*, *Candida*, and *Aspergillus*	Negative	Negative
The CSF Venereal Disease Research Laboratory (VDRL) test	Negative	Negative
CSF *Mycobacterium tuberculosis* smear and culture	Negative	Negative
Serum tests for Wright	1/160	< 1:80
Serum 2‐mercaptoethanol (2 ME)	1/80	< 1:40
Serum Coombs Wright	1/160	< 1:80
Serum tests for rheumatoid factor (RF)	5	< 20 IU/mL
Serum tests for antinuclear antibodies (ANA), VDRL, and human immunodeficiency virus (HIV)	Negative	Negative
The angiotensin‐converting enzyme (ACE) level	9	8–52 U/L
Cytology	Negative for malignant cells	Negative for malignant cells

**FIGURE 1:**
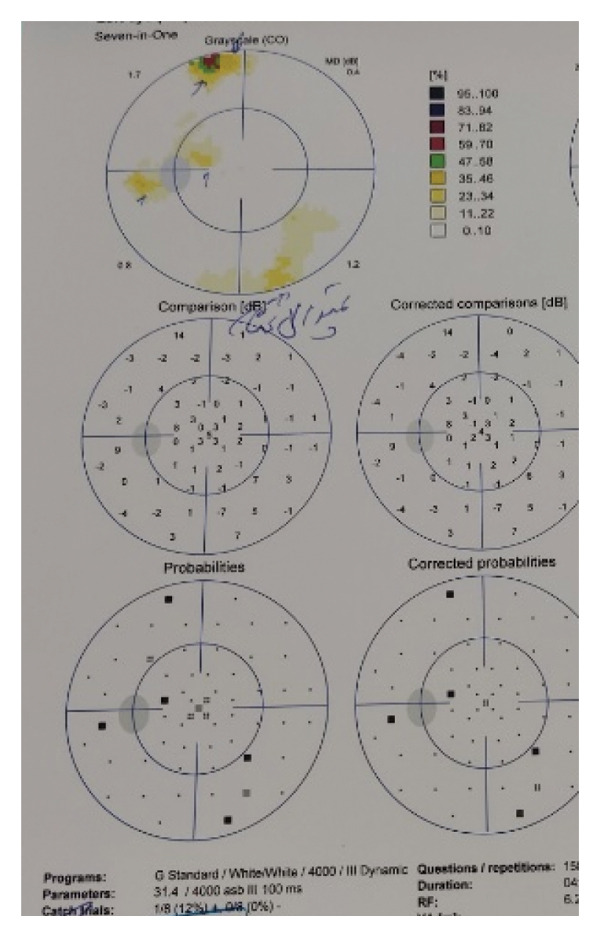
Left eye perimetry: a scotoma in both the superior nasal and central regions.

**FIGURE 2:**
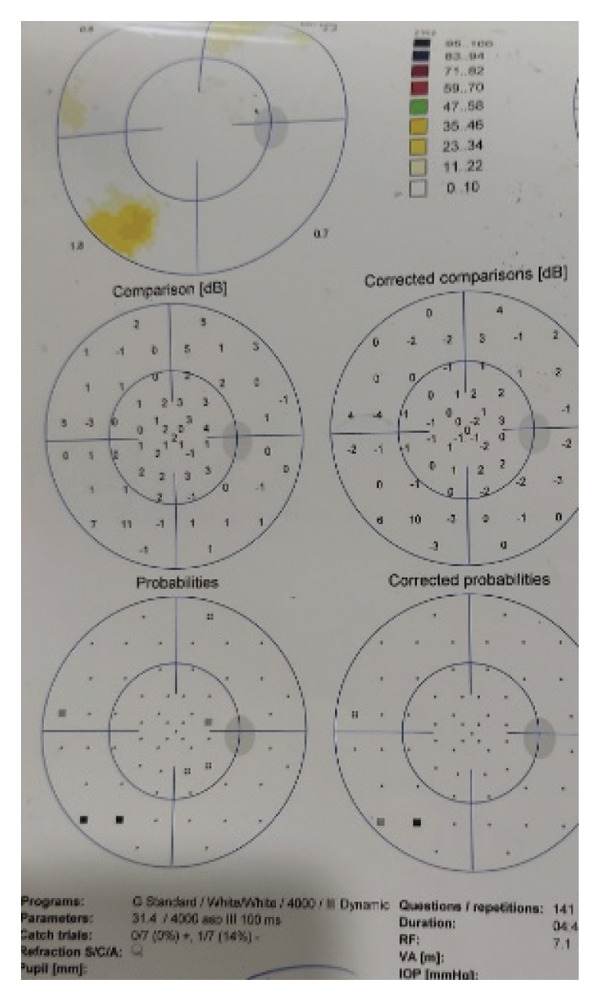
Right eye perimetry: a normal perimeter report for the right eye.

Visual field testing showed a normal perimetric report for the right eye; however, the left eye demonstrated a scotoma in both the superior nasal and central regions (Figures 1 and 2).

Based on the diagnosis of *Brucella* meningitis, the patient was started on a treatment regimen consisting of ceftriaxone 2 g every 12 h, doxycycline 100 mg every 12 h, and rifampin 900 mg daily for 1 month. Following neurology consultation, a 1‐week course of dexamethasone (4 mg every 8 h) was recommended; however, corticosteroid therapy was discontinued after further evaluation due to the absence of optic neuritis.

**FIGURE 3:**
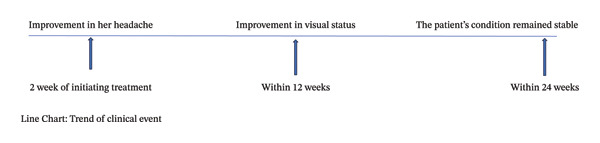
Trend of clinical event.

The patient reported improvement in her headaches within 2 weeks of initiating treatment, followed by gradual improvement in her visual field (Table 2) (see Figure 3).

**TABLE 2:** Timeline of clinical events.

Time/week	Event
Within 2 weeks of initiating treatment	Improvement in her headache
Within 12 weeks of initiating treatment	Improvement in visual status
Within 24 weeks of initiating treatment	The patient’s condition remained stable.

After completing 1 month of therapy with ceftriaxone, doxycycline, and rifampin, the patient was discharged on a three‐drug oral regimen consisting of doxycycline 100 mg every 12 h, rifampin 600 mg daily, and cotrimoxazole (400/80 mg) two tablets every 12 h, to be continued for an additional 5 months.

## 3. Discussion

Subacute and chronic meningitis may arise from a wide range of etiologies, with the most common categories including infectious agents, autoimmune disorders, and neoplasms [7]. Chronic meningitis is defined as inflammation lasting 3 weeks or longer and may result from various infectious causes, including brucellosis. The neurological manifestations of brucellosis are diverse and may include meningitis, meningoencephalitis, encephalitis, cranial neuropathies, increased intracranial pressure, sinus thrombosis, radiculitis, peripheral neuropathy, myelitis, and psychiatric symptoms.

CSF analysis and neuroimaging often lack specificity in NB. CSF abnormalities may include elevated opening pressure, increased protein levels, pleocytosis, and reduced glucose concentration. Therefore, the diagnosis of NB relies on a combination of clinical symptoms, regional epidemiological context, CSF findings—such as *Brucella* agglutination tests, CSF culture, and PCR—and imaging results [8].

A study conducted in Iran between 2007 and 2017 evaluated 54 cases of NB. The most frequently reported symptoms were fever and headache. CSF analysis revealed a median leukocyte count of 75 cells/μL, mean protein levels of 83 mg/dL, and glucose levels of 39 mg/dL. Only two cases demonstrated severe hypoglycorrhachia, and one case showed markedly elevated CSF protein levels exceeding 500 mg/dL [9]. More recent research from China (2024) assessed 21 patients with NB, with a mean age of 40 years. The predominant neurological manifestations included limb weakness (52.38%) and hearing loss (47.62%). The most common nervous system lesions involved the spinal cord (66.67%), cranial nerves (61.90%), central demyelination (28.57%), and meningitis (28.57%). Among patients with cranial nerve involvement, 69.23% had auditory nerve dysfunction, while 15.38% exhibited optic or oculomotor nerve involvement. CSF biochemical analysis was abnormal in all cases [10].

Although the exact incidence of papilledema in NB remains unknown, classic observations by Al Deeb et al. suggest that papilledema—rather than true optic neuritis—is the predominant mechanism of visual disturbance in *Brucella* meningitis, reflecting elevated intracranial pressure [11].

A comprehensive review of more than 30 years of data (1993–2023) highlighted the complexity of NB and emphasized the need for improved diagnostic tools [12]. Additionally, a case report from India described a 41‐year‐old man with a three‐month history of gait disturbance who was ultimately diagnosed with NB through CSF analysis. Despite normal cerebral and spinal MRI findings, elevated *Brucella* antigen titers confirmed the diagnosis, and the patient improved significantly after 3 months of treatment [13].

In the present case, the patient exhibited CSF findings consistent with NB, including elevated opening pressure and mild lymphocytic pleocytosis. She presented with a 2‐month history of headaches beginning 6 weeks’ postpartum and developed unilateral visual disturbances 1 week before evaluation.

Antibiotics remain the cornerstone of NB treatment. Some studies suggest that corticosteroids may help reduce inflammatory responses; however, concerns persist regarding long‐term effects and relapse risk [12]. While corticosteroids are well established for accelerating visual recovery in demyelinating optic neuritis, their role in *Brucella* meningitis–related visual impairment differs fundamentally. Papilledema in NB reflects increased intracranial pressure rather than optic nerve inflammation; thus, corticosteroids are not routinely indicated except in rare cases of true optic neuritis [11].

Future research should aim to refine treatment strategies that address both the direct infectious process and the broader immunological consequences, ultimately improving patient outcomes and quality of life.

On average, patients with NB are approximately 40 years old, with a higher prevalence among males. Most patients report contact with livestock or consumption of unpasteurized dairy products although exposure history may be unclear in some cases. Clinical manifestations are often nonspecific, and the classic triad of meningitis is rarely observed. Mild pleocytosis—defined as fewer than 50 leukocytes per microliter—is common, whereas severe hyperproteinorrhachia and severe hypoglycorrhachia are uncommon. Chronic meningitis typically demonstrates a lymphocytic‐dominant inflammatory pattern although early *tuberculosis* meningitis, *nocardiosis*, or brucellosis may present with neutrophilic predominance [14].

Papilledema has been increasingly recognized in recent NB reports as a consequence of elevated intracranial pressure rather than primary optic nerve inflammation. Visual outcomes are generally favorable with timely antimicrobial therapy, as demonstrated in recent case descriptions of *Brucella* meningitis presenting with bilateral papilledema [15].

Long‐term treatment is essential in NB due to the risk of complications and relapse. Standard therapy typically includes ceftriaxone combined with doxycycline and rifampin, or alternatively cotrimoxazole with doxycycline and rifampin, for a duration of four to 6 months. Overall, prognosis is favorable with appropriate management [16].

In this case, the patient was treated according to established NB guidelines for 6 months using a three‐drug regimen: ceftriaxone 2 g every 12 h for the first month, along with rifampin 900 mg daily and doxycycline 100 mg every 12 h. She was subsequently discharged on doxycycline, rifampin, and cotrimoxazole for an additional 5 months. The patient was closely monitored throughout treatment and follow‐up. Her headaches resolved within 1 week, and her visual field deficits gradually improved. Given the low global prevalence of brucellosis, most available data are derived from case reports. Future research may provide more comprehensive diagnostic and therapeutic strategies.

## 4. Conclusion

Chronic brucellosis meningitis, a form of NB, is a rare but serious complication of *brucellosis* and is characterized by diverse and often nonspecific clinical manifestations. In clinical practice, NB should be considered in the differential diagnosis of patients presenting with neurological symptoms in regions where brucellosis is endemic. Diagnosis relies on a combination of clinical presentation, regional epidemiology, and laboratory findings. Essential diagnostic tools include CSF analysis, *Brucella* agglutination tests, CSF culture, PCR testing, and appropriate neuroimaging studies.

Effective management requires long‐term therapy, as the disease may lead to complications that necessitate close monitoring. Early initiation of combination antimicrobial therapy and adherence to an extended treatment course are critical for achieving favorable outcomes. Although the prognosis is generally positive, antibiotics remain the cornerstone of treatment for NB. Recent interest has emerged regarding the potential role of corticosteroids in reducing inflammatory responses; however, their use should be approached with caution due to the risk of complications and relapse [17–19].

## Author Contributions

Contributor role: Shiva Shabani: data curation, validation, writing–original draft, writing–review and editing, investigation, methodology, and conceptualization.

## Funding

No funding was received for this manuscript.

## Disclosure

No preprint version of this manuscript has been posted. This work has not been presented at any scientific conference or seminar.

## Ethics Statement

This study was approved by the Ethics Committee of Arak University of Medical Sciences (approval no. IR.ARAKMU.REC.1401.291). All procedures involving human participants were conducted in accordance with the ethical standards of the institutional research committee and the Declaration of Helsinki.

## Consent

Informed consent has been taken from the author to publish this report in Journal of Case Reports in Infectious Diseases.

Written informed consent has been obtained from patient to publish this report in accordance with the journal’s patient consent policy.

## Conflicts of Interest

The author declares no conflicts of interest.

## Data Availability

The data used to support the findings of this study are available from the corresponding author upon reasonable request.
